# Tumor Suppressing Subtransferable Candidate 4 Expression Prevents Autophagy-Induced Cell Death Following Temozolomide Treatment in Glioblastoma Cells

**DOI:** 10.3389/fcell.2022.823251

**Published:** 2022-03-02

**Authors:** Yongqiang Chen, Spencer B. Gibson

**Affiliations:** ^1^ CancerCare Manitoba Research Institute, CancerCare Manitoba, University of Manitoba, Winnipeg, MB, Canada; ^2^ Department of Biochemistry and Medical Genetics, Department of Immunology, University of Manitoba, Winnipeg, MB, Canada

**Keywords:** autophagy, autophagy-induced cell death (AuICD), glioblastoma (GBM), temozolomide, TSSC4, LC3-interacting region (LIR), EGFR

## Abstract

Glioblastoma (GBM) is the most common and aggressive type of brain cancer in adults, with temozolomide (TMZ) being widely used as the standard chemotherapy drug for its treatment. However, GBM frequently becomes resistant to TMZ treatment due to various mechanisms including amplification and mutations of the epidermal growth factor receptor (EGFR), where EGFR variant III (EGFRvIII) is the most common EGFR mutation. Autophagy (macroautophagy) is an intracellular “self-degradation” process involving the lysosome. It mainly plays a pro-cell survival role contributing to drug resistance in cancers including GBM, but, under some conditions, it can induce cell death called autophagy-induced cell death (AuICD). We recently published that TSSC4 (tumor suppressing subtransferable candidate 4) is a novel tumor suppressor and a novel autophagy inhibitor that inhibits cancer cell growth through its interacting with the autophagy protein LC3. In this brief research report, we demonstrate that cell death induced by TMZ in GBM cells is inhibited by overexpression of TSSC4. TSSC4 overexpression also prevents TMZ-induced autophagy but not when TSSC4 is mutated in its conserved LC3-interacting region. When EGFRvIII was expressed in GBM cells, TSSC4 protein was increased and TMZ-induced cell death was decreased. Knockout of TSSC4 in EGFRvIII-expressing GBM cells increased TMZ-induced autophagy and cell death. This cell death was decreased by autophagy inhibition, suggesting that TSSC4 downregulation promotes TMZ-induced AuICD. This indicates that TSSC4 is a novel target to sensitize GBM cells to TMZ treatment.

## Introduction

Glioblastoma (GBM) is the most common and aggressive type of brain tumors in adults (Lathia et al., 2015; Singh et al., 2021). A statistical study reported that GBM is the cause of 14.6% of all central nervous system (CNS) tumors and 48.3% of malignant primary brain tumors in the United States in 2012–2016 (Ostrom et al., 2019). An increased rate of GBM was observed from 1995 to 2015 in three countries including England, Canada and the United States (Davis et al., 2020). Although a combination therapy including surgery, radiotherapy and chemotherapy, has been used to treat this disease, a poor outcome remains with the patient survival time of only 15–19 months, which is conferred by drug resistance and the blood-brain barrier (Stupp et al., 2009; Lathia et al., 2015; Stavrovskaya et al., 2016; Singh et al., 2021), where one important mechanism of drug resistance is attributed to EGFR (epidermal growth factor receptor) signaling (Pan and Magge, 2020).

EGFR is a receptor protein that belongs to the epidermal growth factor (EGF) family including EGFR, HER2, HER3 and HER4 (Henson et al., 2017; Pan and Magge, 2020). Dysregulation of EGFR signaling is associated with the development and progression of human cancers (Han and Lo, 2012; Xu et al., 2017). Aberrant or mutant EGFR expression is frequently found in GBM (Xu et al., 2017; Oprita et al., 2021). The most common mutation of EGFR is the EGFR variant type III (EGFRvIII) which leads to the constitutive tyrosine kinase activation of EGFR, present in 25–33% of GBMs and contributing to increased proliferation and drug resistance (Aldape et al., 2004; Cvrljevic et al., 2011).

Studies in recent years have made it clear that autophagy plays an important role in cancer cell growth, proliferation and drug resistance (Mathew et al., 2007; White 2012; Levy et al., 2017; Ariosa et al., 2021). Autophagy is an intracellular catabolic process involving the lysosome. Based on how an intracellular material (cargo) is delivered to lysosome for degradation, autophagy can be classified into three main types including macroautophagy, microautophagy and chaperone-mediated autophagy (Chen and Klionsky, 2011; Cheng et al., 2013; Ariosa et al., 2021). Among these types of autophagy, macroautophagy is the one that has being investigated most extensively and intensively. Thus, we focus on macroautophagy (hereafter referred to as autophagy). During autophagy, a cargo is surrounded by phagophore which will expand and enclose to form the characteristic double-membrane structure autophagosome containing multiple autophagy-related (ATG) proteins including the microtubule-associated protein light chain 3 (LC3). Then, autophagosome will fuse with the lysosome to form autolysosome where cargo is degraded (Chen and Klionsky, 2011; Levy et al., 2017; Ariosa et al., 2021; Chen and Gibson, 2021). Autophagy promotes either cell growth and survival or cell death depending upon the context, playing a double-edge sword in cancer cells (White and DiPaola, 2009; Chen et al., 2016; Ariosa et al., 2021; Chen and Gibson, 2021). The relationship between autophagy and cell death was proposed to include 1) autophagy-associated cell death where autophagy occurs in parallel to apoptosis, 2) autophagy-mediated cell death where autophagy precedes apoptosis, and 3) autophagy-dependent cell death (autophagic cell death) where autophagy causes cell death that is independent of apoptosis or necroptosis (Denton and Kumar, 2019). For the sake of simplicity, we use autophagy-induced cell death (AuICD) to define cell death that is preceded and triggered by autophagy (Chen and Gibson, 2021).

Previous studies have demonstrated that the tyrosine kinase activation of EGFR can inhibit autophagy through the interaction between EGFR and Beclin 1 (Wei et al., 2013; Chen et al., 2016). The molecular mechanisms linking EGFR signaling to other autophagy proteins are still not clear. Our recent study demonstrated that tumor suppressing subtransferable candidate 4 (TSSC4) is upregulated by EGFR activation in GBM cells and TSSC4 acts as a novel autophagy suppressor by interacting with LC3 (Chen et al., 2021), providing an example for linking EGFR signaling to autophagy proteins other than Beclin 1.

The *TSSC4* gene was first isolated in 1999 (Lee et al., 1999). It is located in an imprinted gene domain on chromosome 11p15.5, encoding a predicted protein of 329 amino acids. This imprinted gene domain contains multiple tumor suppressor genes, such as *CDKN1C/p57/KIP2* (Matsuoka et al., 1995), *PHLDA2/TSSC3* (Dai et al., 2012) and *H19* (Hao et al., 1993). TSSC4 expression is associated with Beckwith-Wiedemann syndrome and different types of cancers including Wilms tumor, rhabdoid tumors, rhabdomyosarcoma, and lung, ovarian and breast cancers (Joyce and Schofield, 1998; Lee et al., 1999; Prawitt et al., 2000). The biological functions of TSSC4 have rarely been studied. A recent study reported that TSSC4 is involved in RNA splicing (Klimešová et al., 2021). Our study demonstrated that TSSC4 inhibited cell growth/proliferation *in vitro* and GBM tumor growth *in vivo* by suppressing autophagy (Chen et al., 2021).

The current most effective chemotherapy drug for GBM treatment is temozolomide (TMZ) (Stavrovskaya et al., 2016; Singh et al., 2021). However, TMZ resistance is a major issue in GBM-bearing patients with no response in more than 50% of treated patients (Lee, 2016; Singh et al., 2021). TMZ resistance in GBM can be caused by multiple mechanisms among which autophagy was proposed mainly to be a stimulator for the resistance although some studies have reported the opposite results (Singh et al., 2021).

In this brief research report, we demonstrate that downregulation of TSSC4 promotes TMZ-induced AuICD in GBM cells, suggesting that targeting TSSC4 can be a novel approach for overcoming TMZ resistance in GBM.

## Materials and Methods

### Reagents and Antibodies

Trypan blue solution (T8154), sodium orthovanadate (Na_3_VO_4_) (S6508), 3-methyladenine (3-MA; M9281), chloroquine diphosphate (CQ; C6628), and phosphatase inhibitor cocktails 2 and 3 (P5726, P0044), NP40 (I8896), okadaic acid (O7885), aprotinin (A1153), pepstatin A (P5318), leupeptin (L2884), and phenylmethanesulfonyl fluoride (PMSF) (93482) were purchased from Sigma-Aldrich, and protease inhibitor cocktail (11 836 153 001) from Roche Diagnostics. The siRNAs against *ATG7* (sc-41447), and *Control* siRNA-A (si*Con*) (sc-37007) were purchased from Santa Cruz Biotechnology. The *ATG7* siRNAs are a pool of 3 target-specific 19–25 nt siRNAs.

Primary antibodies: anti-EGFR (2232), anti-ATG7 (8558), anti-LC3B (2775S), and anti-HA (3724) were purchased from Cell Signaling Technology, anti-TSSC4 (sc-136945) from Santa Cruz Biotechnology, and anti-ACTB/actin beta from Sigma-Aldrich (A3853). Secondary antibodies: goat anti-rabbit IgG (H^+^L)-HRP conjugate (170–6515) and goat anti-mouse IgG (H^+^L)-HRP conjugate (170–6516) were obtained from Bio-Rad Laboratories.

### Cell Culture

The GBM cell lines U87, U251, and U251 EGFRvIII, which have been used in our previous publications (Chen et al., 2021), were grown in Gibco DMEM, high glucose medium (Thermo Fisher Scientific, 11965092) supplemented with 100 units of penicillin per mL plus 100 µg of streptomycin per mL (Life Technologies, 15140–122) and 5% fetal bovine serum, in a humidified 5% CO_2_, 37°C incubator. U87 and U251 cell lines were authenticated by Labcorp.

### Western Blot Analysis

Western blot was performed as described in our previous publication (Chen et al., 2021). Total cell lysate (TCL) was obtained by lysis of cells with NP40 protein lysis buffer containing 0.5% NP40, 250 mM NaCl, 50 mM Tris HCl, pH 7.4, 50 mM NaF, 15 mM sodium pyrophosphate, 1 mM glycerophosphate, 1 mM Na_3_VO_4_, 500 nM okadaic acid, 20 μg/ml aprotinin, 0.7 μg/ml pepstatin A, 5 μg/ml leupeptin, 1 mM PMSF, with the addition of protease inhibitor cocktail, phosphatase inhibitor cocktails 2 and 3. The principle of linear range of detection was followed, which means that the quantity range of a protein sample loaded on the SDS-PAGE gel gives a linear relationship between the amount of total proteins and the band intensity of a target protein on the membrane transferred from the gel (Taylor et al., 2013). In this study, the combined linear range of detection was applied to detect a target protein (for example, LC3B-II) by using an amount of total proteins that produces signals on transfer membrane within a linear range of detection both for a target protein and the housekeeping protein ACTB. Using our system, we found that a range of 2–40 µg of total proteins made all proteins (including ACTB) (from different cell lines) tested to fit in a linear range of detection. The western blot data were generated by using 10–40 µg total proteins. ImageJ program was used to quantify the intensity of protein bands.

### Measurement of Autophagy

During autophagy, the cytosolic form of LC3, LC3-I, is converted to its lipidated form, LC3-II, on autophagosome membranes. Then, LC3-II on the inner membrane of autophagosomes will be degraded in the autolysosome, whereas LC3-II on the outer membrane of autophagosomes will be delipidated byATG4 to become LC3-I when it is relocated on autolysosomes. Since autophagy is a dynamic process, functional autophagy has to be determined by measuring autophagic flux. Autophagic flux can be measured by quantifying the amount of LC3-II or the autophagy substrate SQSTM1/p62 in the absence and presence of a lysosomal inhibitor, via western blot or microscopy. A high level of LC3-II or SQSTM1/p62 in the presence of a lysosomal inhibitor compared to that in the absence of such an inhibitor indicates a positive autophagic flux and therefore functional autophagy. Under functional autophagy, levels of autophagy can be determined by comparing the LC3-II and SQSTM1/p62 levels in the presence of a lysosomal inhibitor. In our previous study, the role of TSSC4 in autophagy has been demonstrated by measuring autophagic flux via western blot of LC3B-II and SQSTM1/p62, and fluorescent microscopy of mRFP-LC3 puncta, in the absence and presence of the lysosomal inhibitor chloroquine (CQ), where consistent results have been obtained through all of these methods (Chen et al., 2021). Since this brief research report is the continuous work of this study (Chen et al., 2021), autophagic flux was measured only by using western blot of LC3B-II in the absence and presence of CQ (20 µM). When the autophagy inhibitor 3-methyladenine (3-MA) was used, cells were pre-incubated with it for 1 h before treatment.

When cells were treated with starvation of glucose and glutamine (No GP), growth medium was removed and cells were incubated with No GP medium which was made by using the Dulbecco’s Modified Eagle’s Medium without glucose, l-glutamine, phenol red, sodium pyruvate and sodium bicarbonate (Sigma-Aldrich, D5030).

### Generation of *TSSC4* Knockout Cell Line Using CRISPR-Cas9 Gene Editing

The double nickase Clustered Regularly Interspaced Short Palindromic Repeats (CRISPR)-CRISPR associated protein 9 (CRISPR-Cas9) plasmids designed by Santa Cruz Biotechnology were used to knock out *TSSC4* in cells as described in our publication (Chen et al., 2021). Briefly, TSSC4 double nickase plasmid (sc-410490-NIC) (NIC-*TSSC4*) and the control double nickase plasmid (sc-437281) (NIC-*Con*) were transfected into U251EGFRvIII cells using Lipofectamine 2000. Then, stable cell lines were developed with puromycin selection followed by single colony pickup. *TSSC4* knockout was verified by western blot.

### Development of Stable Cell Lines

Human TSSC4 plasmid (EX-Z7596-M45) (TSSC4-3xHA/TSSC4) and it empty control vector (EX-NEG-M45) (3xHA/Vector) were purchased from GeneCopoeia. The TSSC4 mutant (F_97_A-D_98_-C_99_-L_100_A) (TSSC4M-3xHA/TSSC4M) was generated by site-directed mutagenesis using the Phusion Site-Directed Mutagenesis Kit (F541) from Thermo Fisher Scientific. Amino acid replacements were confirmed by DNA sequencing and off-target mutations were not found in the open reading frame (ORF) of *TSSC4*. These plasmids were transfected into U87 cells using Lipofectamine 2000 and stable cells were generated by treating cells with G418 (geneticin; Thermo Fisher Scientific, 11811023) (Chen et al., 2021).

### Analysis of Cell Death by Trypan Blue Exclusion Assay

Cell death was measured by flow cytometry as previously described (Chen et al., 2009) or by counting the percentage of dead cells after trypan blue staining on a DeNOVIX CellDrop FL fluorescence cell counter. Trypan blue is excluded from live cells but penetrates into dead cells giving a red fluorescence (Strober, 2015) that can be quantified by flow cytometry or an automated cell counter.

### Statistical Analysis

All data represent at least 2–3 independent experiments. For cell death assay, 4–6 replicates experiments were performed. Data were presented as means ± standard deviation (SD) (n≥3). The Student *t* test with two-tailed distribution and unequal variances was performed for statistical analysis. A value of *p* < 0.05 is considered to be statistically significant. *p < 0.5; **p < 0.01; ***p < 0.001.

## Results

### TSSC4 is Expressed in GBM

**FIGURE 1:**
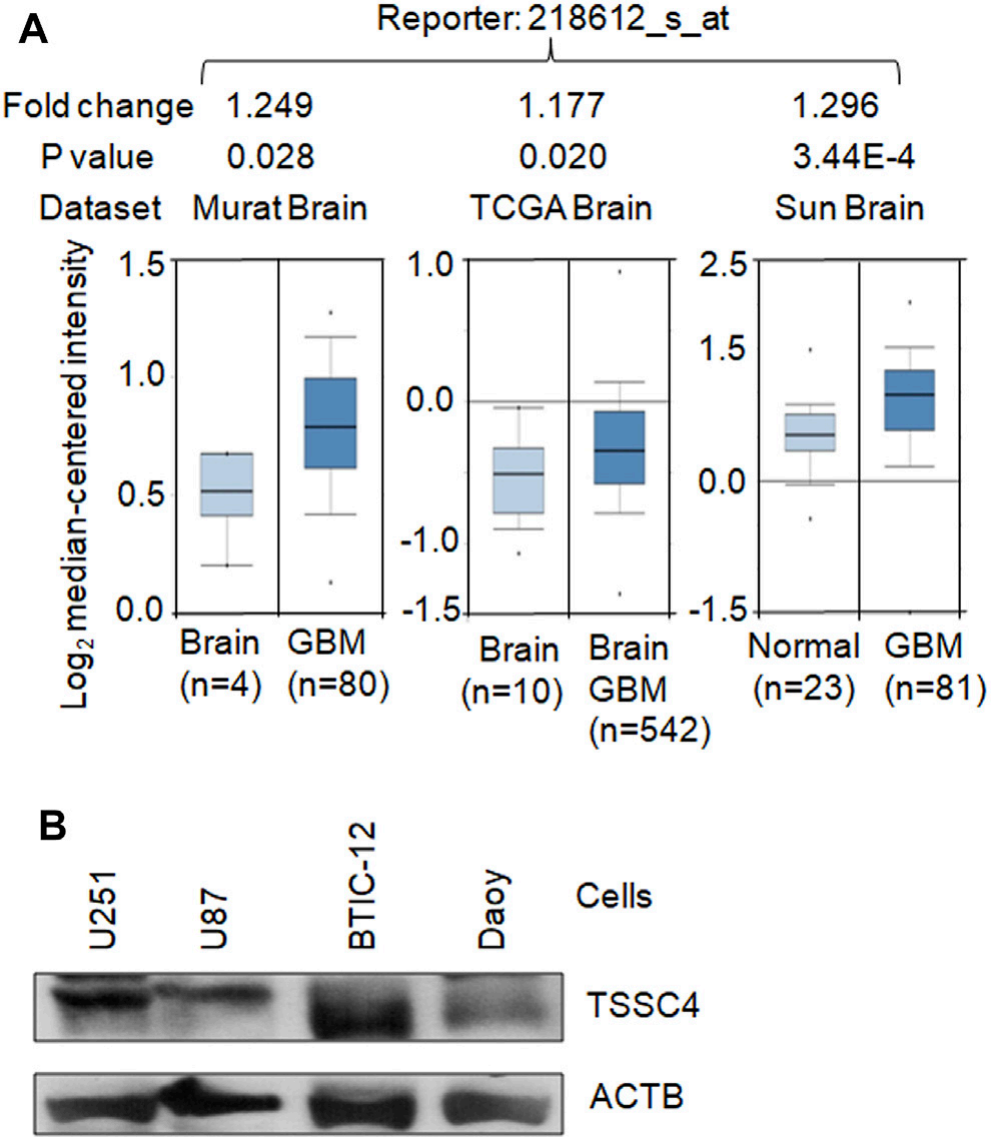
TSSC4 is expressed in GBM. **(A)** TSSC4 is up-regulated in human GBM tissues compared to that in the normal brain tissues. The mRNA microarray data graphs of the relative expression levels of *TSSC4* in GBM tissues against their corresponding normal brain tissues were downloaded from three Oncomine datasets (https://www.oncomine.org). Fold change was calculated based on the values of “log2 median-centered intensity.” Statistical significance is indicated by *p* < 0.05. **(B)** TSSC4 protein is expressed in different types of brain cancer cells. Protein extracts from different cancer cell lines were western blotted and actin beta (ACTB) was used as a loading control. Classification of cell lines: U251 and U87, glioblastoma; BTIC-12, brain tumor initiating cells; Daoy, medulloblastoma.

As demonstrated in our recent publication (Chen et al., 2021), TSSC4 is expressed in GBM tissues and cells. Three Oncomine datasets (https://www.oncomine.org) report higher levels of *TSSC4* mRNA in GBM tissues compared to that in normal brain tissues (Figure 1A). The TSSC4 protein is expressed in the U251 and U87 GBM cell lines, the BTIC-12 brain tumor initiating cells, and the Daoy medulloblastoma cell line (Figure 1B) (Chen et al., 2021).

### Expression of EGFRvIII Increases TSSC4 Expression and Inhibits TMZ-Induced Cell Death in GBM Cells

**FIGURE 2:**
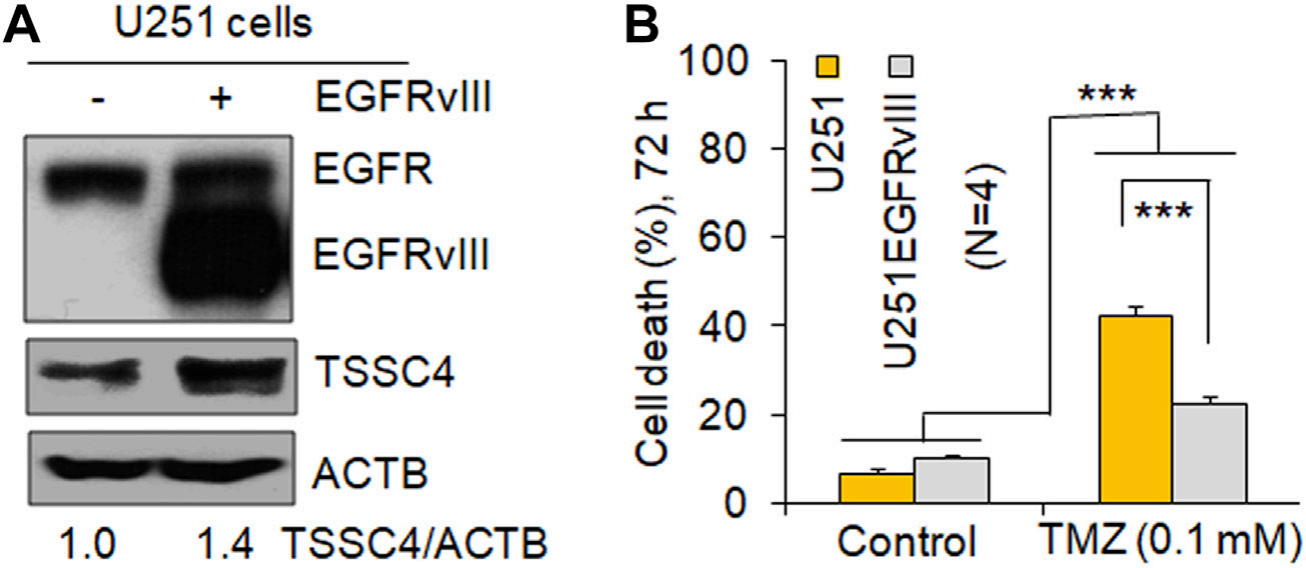
Expression of EGFRvIII increases TSSC4 expression and inhibits TMZ-induced cell death in GBM cells. **(A)** Expression of EGFRvIII increases TSSC4 protein expression in U251 cells. **(B)** Effect of EGFRvIII expression on temozolomide (TMZ)-induced cell death in U251 cells. Cell death was measured by counting the percentage of dead cells after trypan blue staining on a DeNOVIX CellDrop FL fluorescence cell counter. *p < 0.5; **p < 0.01; ***p < 0.001. ACTB was used as a loading control.

EGFR overexpression and/or hyperactivation is frequently observed in human cancers including GBM (Xu et al., 2017) and EGFR alterations are used as a prognostic marker for GBM and contribute to EGFR activation-induced drug resistance in GBM (Oprita et al., 2021). It is well known that expression of the EGFR mutant EGFRvIII constitutively activation EGFR (Oprita et al., 2021; Chen et al., 2021). Similar to our previous report (Chen et al., 2021), EGFRvIII expression increased TSSC4 protein by 40% in U251 cells (Figure 2A). In U251 cells, EGFRvIII expression inhibited TMZ-induced cell death by more than 45% (Figure 2E), consistent with the known feature of EGFR activation as an inducer of drug resistance in GBM (Oprita et al., 2021). However, the underlying mechanisms are still not clear.

### TSSC4 Inhibits TMZ-Induced Autophagy in GBM Cells

To test whether TSSC4 contributes to EGFRvIII-induced drug resistance in GBM cells, we examined TSSC4 regulation of autophagy in the absence and presence of TMZ in GBM cells.

**FIGURE 3:**
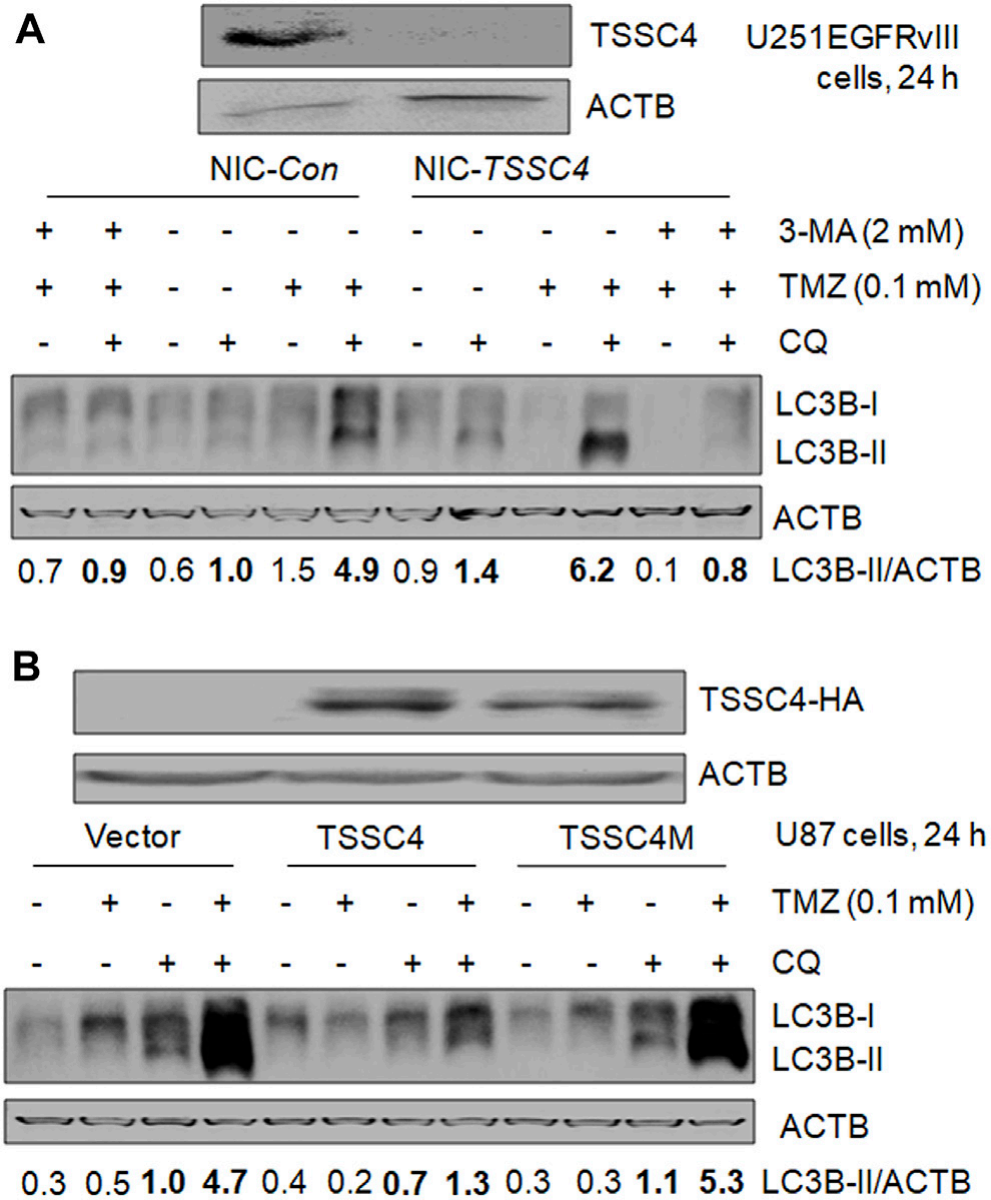
TSSC4 regulates autophagy in GBM cells. Autophagy was measured by western blot of LC3B-II. Autophagic flux was measured by using the lysosomal inhibitor chloroquine (CQ, 20 µM). ACTB was used as a loading control. *NIC-Con*, control cells; *NIC-TSSC4*, TSSC4 knockout cells. **(A)** Knockout of TSSC4 increased basal autophagy and TMZ-induced autophagy in U251EGFRvIII cells following 24 h treatment. The autophagy inhibitor 3-methyladenine (3-MA) was added 1 h before TMZ and CQ. **(B)** Overexpression of TSSC4 but not TSSC4M inhibited basal autophagy and TMZ-induced autophagy in U87 cells following 24 h treatment. Expression of TSSC4(-3xHA) and TSSC4M(-3xHA) was detected by using HA tag antibody. TSSC4M is a mutant of TSSC4, where F_97_ and L_100_in the conserved LIR (LC3-interacting region) (F_97_-D_98_-C_99_-L_100_) of human TSSC4 were replaced by alanine. All experiments represent at least three independent experiments. *p < 0.5; **p < 0.01; ***p < 0.001.

In our recent publication, we have demonstrated TSSC4 inhibition of autophagy by using different methods in autophagy analysis including western blot of LC3B-II and SQSTM1/p62, and fluorescent microscopy of mRFP-LC3 puncta, which generated consistent results (Chen et al., 2021). Hence, in this brief research report, we only used western blot of LC3B-II in the absence and presence of the lysosomal inhibitor chloroquine (CQ) to measure autophagy. Autophagy was measured in control (NIC-*Con*) and *TSSC4* knockout (NIC-*TSSC4*) U251 cells stably expressing EGFRvIII, without and with TMZ treatment for 24 h. In the NIC-*Con* cells, the addition of CQ increased the relative levels of LC3B-II protein from 0.6 to 1.0 and from 1.5 to 4.9 without and with TMZ, respectively; and in the NIC-*TSSC4* cells, the addition of CQ increased the relative levels of LC3B-II protein from 0.9 to 1.4 and from less than 0.1 to 6.2 without and with TMZ, respectively (Figure 3A). Furthermore, the autophagy inhibitor 3-methyladenine (3-MA) significantly decreases LC3B-II levels in NIC-*Con* and NIC-*TSSC4* cells, in the presence of CQ and TMZ (Figure 3A), supporting that 3-MA inhibits TMZ-induced autophagy in control and *TSSC4* knockout cells. The same trends of LC3B-II changes were observed in two more independent experiments by treatment of cells for 24 and 72 h, respectively (data not shown). These results support that 1) a positive autophagic flux and therefore functional autophagy exist in the control and *TSSC4* knockout cells without and with TMZ treatment, 2) TMZ increases autophagy in control and *TSSC4* knockout cells, and 3) TSSC4 knockout increases basal autophagy (without TMZ) and TMZ-induced autophagy (with TMZ), consistent with our recent report (Chen et al., 2021).

In our recent publication, we created the mutant TSSC4 plasmid called TSSC4M by changing F_97_ and L_100_ to alanine in the conserved LC3-interacting region (LIR) (FDCL) of human *TSSC4* cDNA-encoded TSSC4 protein and demonstrated that TSSC4 inhibits autophagy by interacting with LC3-I via its conserved LIR and TSSC4M does not inhibit autophagy due to the destruction of its interaction with LC3 (Chen et al., 2021). We measured the effects of TSSC4 and TSSC4M overexpression on TMZ-induced autophagy in U87 cells.

In cells expressing vector alone, TSSC4 or TSSC4M, CQ addition led to a higher level of LC3B-II with and without TMZ, respectively, and in the presence of CQ, TMZ addition increased LC3B-II in each type of cells (Figure 3B), indicating that functional autophagy exists in the absence and presence of TMZ and TMZ promotes autophagy in all three types of cells but much less pronounced in TSSC4 overexpressed cells. When cells were treated for 24 h, in the presence of CQ, overexpression of TSSC4 decreased LC3B-II level from 1.0 to 0.7 in the absence of TMZ and from 4.7 to at least 1.3 in the presence of TMZ, whereas overexpression of TSSC4M did not decrease the level of LC3B-II in the absence or presence of TMZ (Figure 3B). The same trends of LC3B-II changes were observed in two more independent experiments by treatment of cells for 24 and 72 h, respectively (data not shown). These results suggest that overexpression of TSSC4 but not TSSC4M inhibits basal autophagy and TMZ-induced autophagy and further support TMZ increases autophagy in GBM cells. Therefore, TSSC4 inhibits basal autophagy and TMZ-induced autophagy through its conserved LIR.

### TSSC4 Inhibits Autophagy-Induced Cell Death (AuICD) in GBM Cells Treated by TMZ

**FIGURE 4:**
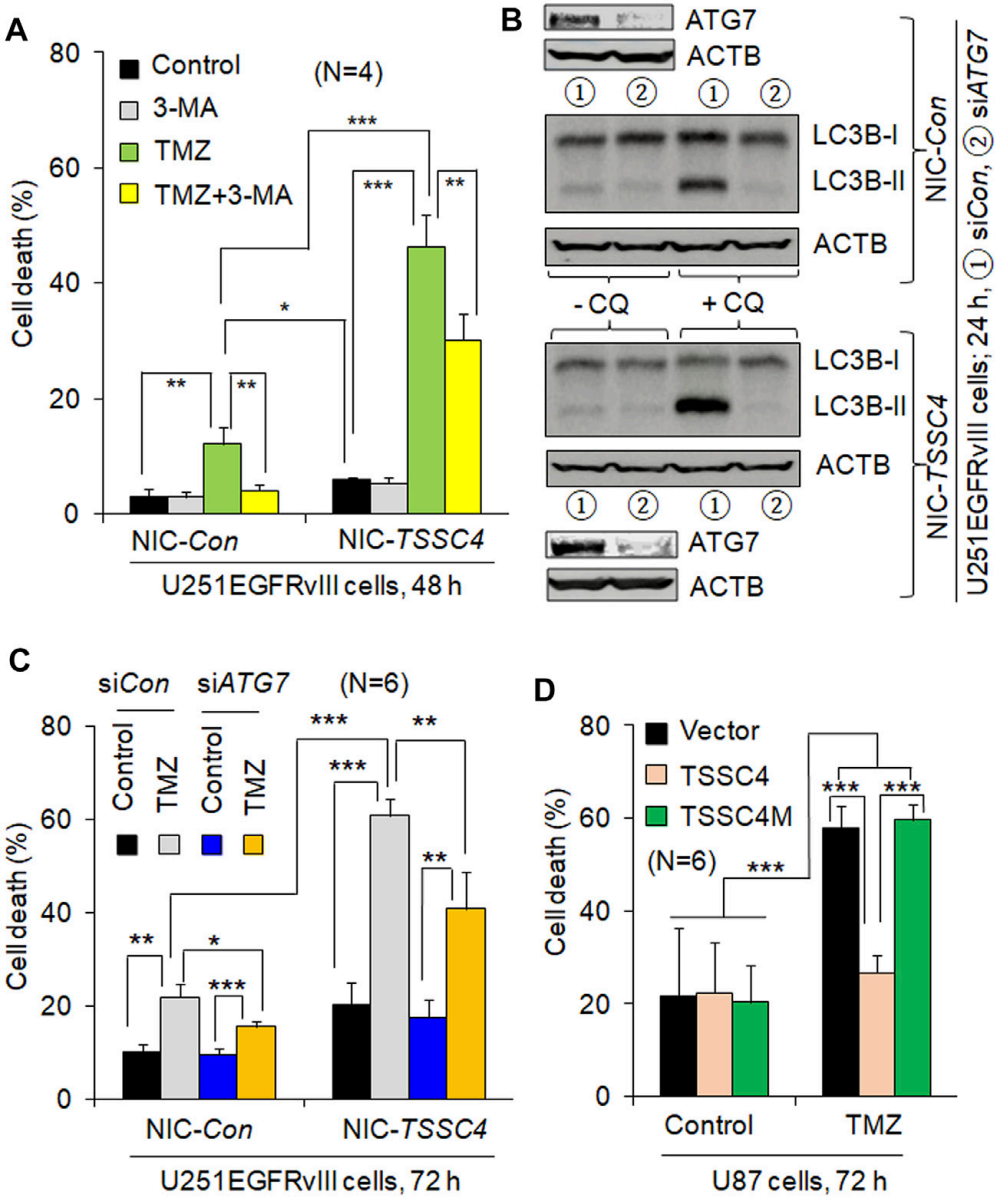
TSSC4 regulates TMZ-induced cell death in GBM cells. Cell death was measured by flow cytometry after cells were stained by trypan blue. *NIC-Con*, control cells; *NIC-TSSC4*, TSSC4 knockout cells. **(A)** Knockout of TSSC4 increased TMZ-induced cell death and 3-MA inhibited TMZ-induced cell death in U251EGFRvIII cells. Cells were pre-incubated with 3-MA (2 mM) for 1 h before the addition of TMZ (0.1 mM, the same hereafter). TSSC4 knockout was demonstrated in Figure 3A. **(B)** Western blot demonstration of *ATG7* knockdown by siRNA silencing and its effect on autophagy in *NIC-Con* and *NIC-TSSC4* U251EGFRvIII cells. Autophagy was measured by western blot of LC3B-II in the absence and presence of chloroquine (CQ, 20 µM). ACTB was used as a loading control. **(C)** Knockdown of *ATG7* inhibited TMZ-induced cell death in *NIC-Con* and *NIC-TSSC4* U251EGFRvIII cells. **(D)** Overexpression of TSSC4 but not TSSC4M inhibited TMZ-induced cell death in U87 cells. Expression of TSSC4 and TSSC4M was demonstrated in Figure 3B. All experiments represent at least three independent experiments. *p < 0.5; **p < 0.01; ***p < 0.001.

To examine whether TSSC4-mediated autophagy regulates cell death, we measured the effects of autophagy inhibition and elevation on TMZ-induced cell death in GBM cells expressing different levels of TSSC4. In U251EGFRvIII cells, TMZ treatment caused 12 and 46% cell death at 48 h (Figure 4A) and 22 and 60% at 72 h (Figure 4C) in control (NIC-*Con*) and *TSSC4* knockout (NIC-*TSSC4*) cells, respectively, indicating that TSSC4 knockout increased TMZ-induced cell death. The autophagy inhibitor 3-MA inhibited TMZ-induced cell death by at least 30% in control and *TSSC4* knockout cells at 48 h, respectively (Figure 4A). Knockdown of the autophagy gene *ATG7* by siRNA silencing decreased autophagy, which was measured by western blot of LC3B-II in the absence and presence of CQ, in control and *TSSC4* knockout cells, respectively (Figure 4B). TMZ-induced cell death was inhibited by *ATG7* siRNA by at least 20% in Control and *TSSC4* knockout cells (Figure 4C), respectively. This experiment was repeated by another experiment with control and *TSSC4* knockout U251 cells and by the third experiment with *TSSC4* knockout cells, resulting in the same observations (data not shown). These results suggest that TMZ induces autophagy-induced cell death (AuICD) which is increased by TSSC4 knockout in GBM cells. This is further supported by the observation that overexpression of TSSC4 but not TSSC4M inhibited TMZ-induced cell death in U87 cells (Figure 4D). Thus, TSSC4 inhibits AuICD induced by TMZ through suppressing autophagy in GBM cells.

## Discussion

TMZ is the standard therapy for GBM but it frequently leads to tumor drug resistance and tumor recurrence which mechanisms are still not clear (Singh et al., 2021). In this brief research report, we demonstrate that TSSC4 contributes to drug resistance by inhibiting TMZ-induced cell death in EGFRvIII-expressing GBM cells via autophagy suppression. Activation of EGFR signaling is a frequent event in GBM, where EGFRvIII is the most common activation mutation of EGFR, contributing to drug resistance (Oprita et al., 2021). EGFRvIII increases TSSC4 expression (Figure 2) (Chen et al., 2021) although TSSC4 inhibits proliferation and tumor growth of cancer cells with increased activation of EGFR signaling (Chen et al., 2021), generating a paradox that cancer cells upregulate a protein to suppress their proliferation and tumor growth. The finding that TSSC4 expression can convey a drug resistance mechanism provides a reason for its up-regulation by EGFR signaling.

Autophagy mainly supports cell survival but induces cancer cell death under some conditions (Chen and Gibson, 2021). Our previous study demonstrated that a low level of autophagy supports cell survival at an early time of hypoxia, whereas autophagy was enhanced to induce autosis (a type of AuICD) at a later time of hypoxia (Chen et al., 2016). In this study, we demonstrate that TMZ increases autophagy, which is inhibited by 3-methyladenine (3-MA), an autophagy inhibitor that targets the Beclin 1-Vps34 complex upstream of the autophagy pathway. This is consistent with the report in the literature (Li et al., 2019). TMZ induces apoptosis-independent cell death in GBM cells (Kanzawa et al., 2004). Interestingly, TMZ-induced cell death was inhibited by 3-MA, supporting TMZ induced AuICD. In contrast, TMZ-induced cell death was increased by bafilomycin A1, an autophagy inhibitor and a lysosomal inhibitor targeting the degradation step in the autolysosomes downstream of the autophagy pathway. This shifted cell death to apoptosis (Kanzawa et al., 2004). Other studies also reported that TMZ-induced cell death was increased by the lysosomal inhibitors chloroquine (CQ) and bafilomycin A1 (Golden et al., 2014; Hori et al., 2015). However, it was reported that the effect of CQ on cancer cell death may be independent of its effect on autophagy (Maycotte et al., 2012). We demonstrated that TMZ-induced cell death was reduced by 3-MA or knockdown of *ATG7*, an essential component for autophagosome formation downstream of the autophagy pathway, in control and *TSSC4* knockout GBM cells and *TSSC4* knockout increases TMZ-induced cell death, supporting that TMZ induces AuICD which can be elevated by downregulation of TSSC4. Similarly, TMZ induced AuICD and the microRNA miR-519a promoted TMZ-induced autophagy leading to increased AuICD in a TMZ-resistant U87 cell line, (Li et al., 2018). In the future, we will verify TMZ-induced AuICD in more types of GBM cells and investigate the roles of TSSC4 in cell death in different types of cancer cells with diverse genetic backgrounds.

Taken together, we demonstrated that downregulation of TSSC4 can increase autophagy and AuICD in GBM cells treated with TMZ, providing a novel strategy to overcome TMZ-induced drug resistance by targeting TSSC4 and autophagy.

## Data Availability

The original contributions presented in the study are included in the article/Supplementary Material, further inquiries can be directed to the corresponding author.
